# Gender-aware Parkinson’s care: a design-based study of patient perspectives on gender norms and gender-sensitive care

**DOI:** 10.1016/j.eclinm.2023.102285

**Published:** 2023-10-17

**Authors:** Irene Göttgens, Linda Modderkolk, Paula Vermuë, Sirwan K.L. Darweesh, Bastiaan R. Bloem, Sabine Oertelt-Prigione

**Affiliations:** a Department of Primary and Community Care, Radboud Institute for Health Sciences, Radboud University Medical Centre, Nijmegen, the Netherlands; b Department of Neurology, Centre of Expertise for Parkinson & Movement Disorders, Donders Institute for Brain, Cognition and Behaviour, Radboud University Medical Centre, Nijmegen, the Netherlands; c AG 10 Sex- and Gender-sensitive Medicine, Medical Faculty OWL, University of Bielefeld, Bielefeld, Germany

**Keywords:** Parkinson’s disease, Gender norms, Gender awareness, Design-based methodology

## Abstract

**Background:**

Gender dimensions are progressively recognised as a relevant social determinant of health in people with Parkinson’s disease (PD). However, little is known about the impact of gender norms and stereotypes on illness experiences of men and women with PD and what they consider important focal points for gender-sensitive PD care.

**Methods:**

We conducted two equity-centred design (ECD) sessions on December 7, 2022 and December 8, 2022, at the Radboud University Medical Centre in the Netherlands. This participatory multi-method approach includes patients in the research and design process and was used to explore the impact of gender norms and stereotypes in illness experiences and generate patient-driven recommendations for gender-aware Parkinson’s care. Quantitative survey data and design-based data were descriptively analysed, and qualitative focus group discussions were thematically analysed.

**Findings:**

This study included thirteen men and fifteen women with PD in the Netherlands. All participants were of Dutch descent, with a median age of 65.5 years and a median clinical disease duration of 4.2 years. The gendered stereotype that “*people with PD are old men*” affected both men’s and women’s perception of living with the disease and the perceptions of their social environment. Men described masculine stereotypes related to physical strength and provider roles, while women expressed those related to feminine physical appearance and caregiver roles, influencing their illness experiences. For some, these norms influenced personal behaviours, while for others, they affected experiences through societal attitudes.

**Interpretation:**

Our findings suggested that several gender norms and stereotypes influence the illness experiences of men and women with PD, manifesting at ideological, interpersonal, and internalised levels. Some participants internalised these norms, affecting their coping behaviours, while others encountered them in broader ideological contexts that shaped societal attitudes and interpersonal relationships. To advance gender sensitive PD care, it's essential to explore the impact of gender roles and norms, especially regarding how they might impede coping strategies, care access and utilisation for individuals of diverse gender identities.

**Funding:**

The Gatsby Foundation and co-funded by the PPP Allowance by 10.13039/100016036Health∼Holland. Travel reimbursements for participants were made available through a 10.13039/100013301Parkinson's Foundation grant (PF-FBS-2026).

**Research in context**

**Evidence before this study**

The authors reviewed publications in the peer-reviewed literature that addressed the impact of gender norms and stereotypes on health in general and in the context of Parkinson’s disease in particular. The search was performed in PubMed, PsyINFO and Web of Science between 15 February 2023 and 5 May 2023, the time frame of the search was not limited, and search queries included: ((Gender[Ti] OR "Gender∗ role∗" [Ti] OR "Gender∗ norm∗" [Ti] OR "Gender∗ stereotype∗" [Ti] OR "Gender∗ relation∗" [Ti] OR (feminin∗[Ti] OR masculin∗[Ti]))) and ((Parkinson disease [MeSH Terms]) OR (Parkinson∗[Ti])) For non-medical databases (health[Ti] OR clinic[Ti] OR medic∗[Ti]) was added.

**Added value of this study**

This study fills a critical knowledge gap by investigating the role of gender norms and stereotypes in illness experiences of men and women with Parkinson's disease (PD), an area that has remained relatively unexplored. Through an innovative equity-centred design approach that actively involves men and women with PD, the research sheds light on how prevailing gender norms and stereotypes, including the gendered perception that '*People with PD are old men*', shape the illness experiences of both men and women living PD. The study highlights specific areas for gender-sensitive PD care, including addressing personality changes, emotional experiences, and navigating gender-specific norms that can impede or facilitate access to care. This research underscores the importance of recognising the nuanced ways in which gender norms and stereotypes can impact illness experiences.

**Implications of all the available evidence**

Recommendations for gender-sensitive PD care and research include fostering awareness among researchers and healthcare providers about gender norms and stereotypes to avoid the reproduction of gender biases in research and clinical settings as well as the encouragement to proactively address social role changes due to the progression of PD related symptoms.

## Introduction

There is emerging evidence of Parkinson disease (PD) is the fastest growing neurological disorder worldwide. The global number of people with PD is projected to exceed 12 million by 2040.^1^ Due to the chronic and progressive nature of PD, the development of interventions that can delay disability and enable people with PD to continue participating in activities and social roles is considered a key priority in PD research.^2^

Gender is progressively recognised as a relevant social determinant of health in people with PD, but its multidimensional operationalisation in biomedical research is challenging.^3^ A recent study demonstrated that different gender dimensions, such as gender identity, gendered social norms, roles and relations, have a heterogenous impact on the quality of life of people with PD, emphasising the importance of a precise study of distinct gender dimensions in the context of PD.^4^ In fact, previous research has shown that the adherence to traditional gender norms, and their related roles, is a stronger predictor of health outcomes than gender identity, and that norms related to gender influence the illness experiences of people with PD.^4^, ^5^, ^6^, ^7^

Gender norms include cultural beliefs and expectations about how people with different gender identities should act and interact with each other, and are part of a gendered socialisation and stereotyping process.^8^ Investigating gender norms in healthcare settings, thus, focuses on the presence of social expectations and beliefs attributed to gender that can affect health and health outcomes among particular patient populations. Given the impact of gender norms and expectations on social roles, investigating the particular role of gender norms in illness experiences of people with PD is imperative.^5^

Understanding how gender norms affect health and illness experiences of people with PD is essential for the design of programs and policies that combat harmful norms and improve gender equality in health.^9^^,^^10^ However, a recent review of health interventions that addressed detrimental gendered norms and stereotypes concluded that longitudinal data on their effectiveness is currently lacking.^11^ The review reported that most of the initiatives designed to shift attitudes and behaviours regarding gendered stereotypes and norms rely too much on self-reported survey data that may be subjected to social desirability bias and the use of participant observations and key informant feedback would strengthen evaluations. Accurately capturing the impact of gender norms on health is a complex matter as these social norms are dependent on socio-cultural context, time and place.^12^ Surveys used in biomedical research often include proxy measures to investigate how gender norms can affect health but they might lack specificity to capture the full range of gender norms and stereotypes that exists in a particular context and often ask about gender norms in binary terms (men/women), thereby excluding the experiences of people who identify otherwise. The study of these contextual gender norms and stereotypes requires multimethod approaches that enable the investigation of the complex relationship between gender identities, contextual gender norms and roles, and their impact on individual health.^11^

Because much of the existing knowledge regarding gender norms and stereotypes has arisen from research conducted in general population settings, there is a lack of understanding how gender norms and stereotypes unfold within a healthcare context and particularly, whether and how they influence health and care experiences of men and women with PD. Therefore, this study aims to 1) understand the role of stereotypical gender norms in the illness experiences of men and women with PD; and 2) ideate focal points for gender-sensitive PD care from patients’ perspectives, using an equity-centred design approach.

## Methods

### Equity-centred design approach

**Fig. 1:**
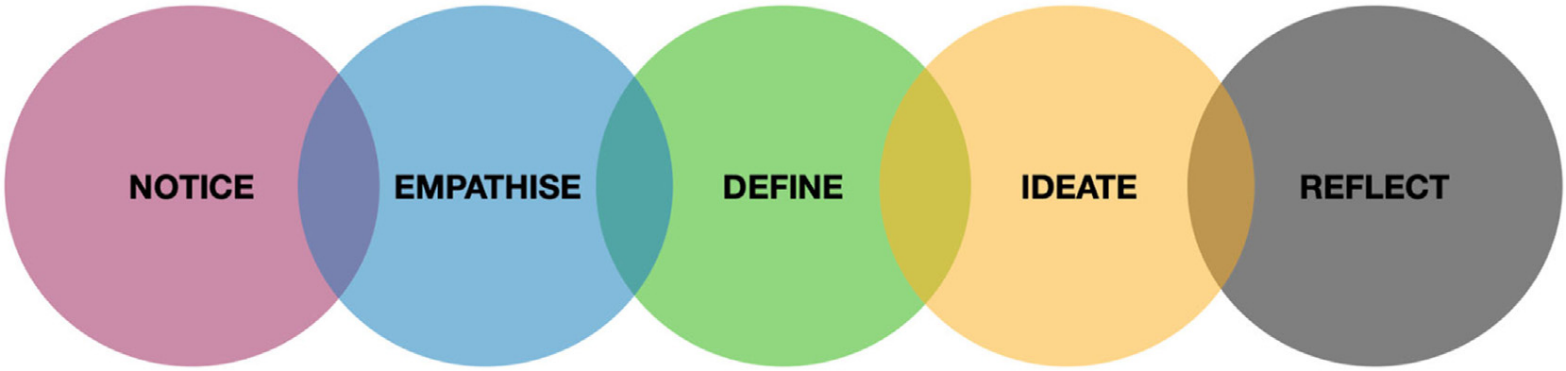
**Adapted equity-centered design framework from Raz et al. (2017), as applied in this study.** Colour Cade: Circles are Pink, Blue, Green, Yellow, Grey. Text is black.

This design-based study was embedded in the PRIME-NL Study, a large cohort study in the Netherlands focused on PRoactive and Integrated Management and Empowerment in Parkinson’s Disease.^13^ An equity-centred design approach inspired by Raz and Clifford (2017) was used to guide the research and design process, focusing on the first three phases of the process, including the notice and reflect phase (Fig. 1).^14^ Equity-centred design builds on the human-centred design framework by incorporating intentional reflectivity and acknowledge power, identity and context in which the design process takes place. Participatory design sessions were organised for men and women separately and on two different days; one workshop day for men with PD and one workshop day for women with PD (for details, see Supplement 1). The reporting is guided by the guidelines for reporting health research involving design, by Bazzano et al. (2020) (Supplement 2) and by the SAGER guidelines.^15^^,^^16^

### Ethics statement

This study has been assessed by the Ethical Board of the Radboud University Medical Centre (METC Oost-Nederland, file number 2022-15954). All participants signed an informed consent at the start of the workshop.

### Theoretical framework for gender norms and gender stereotypes

**Fig. 2:**
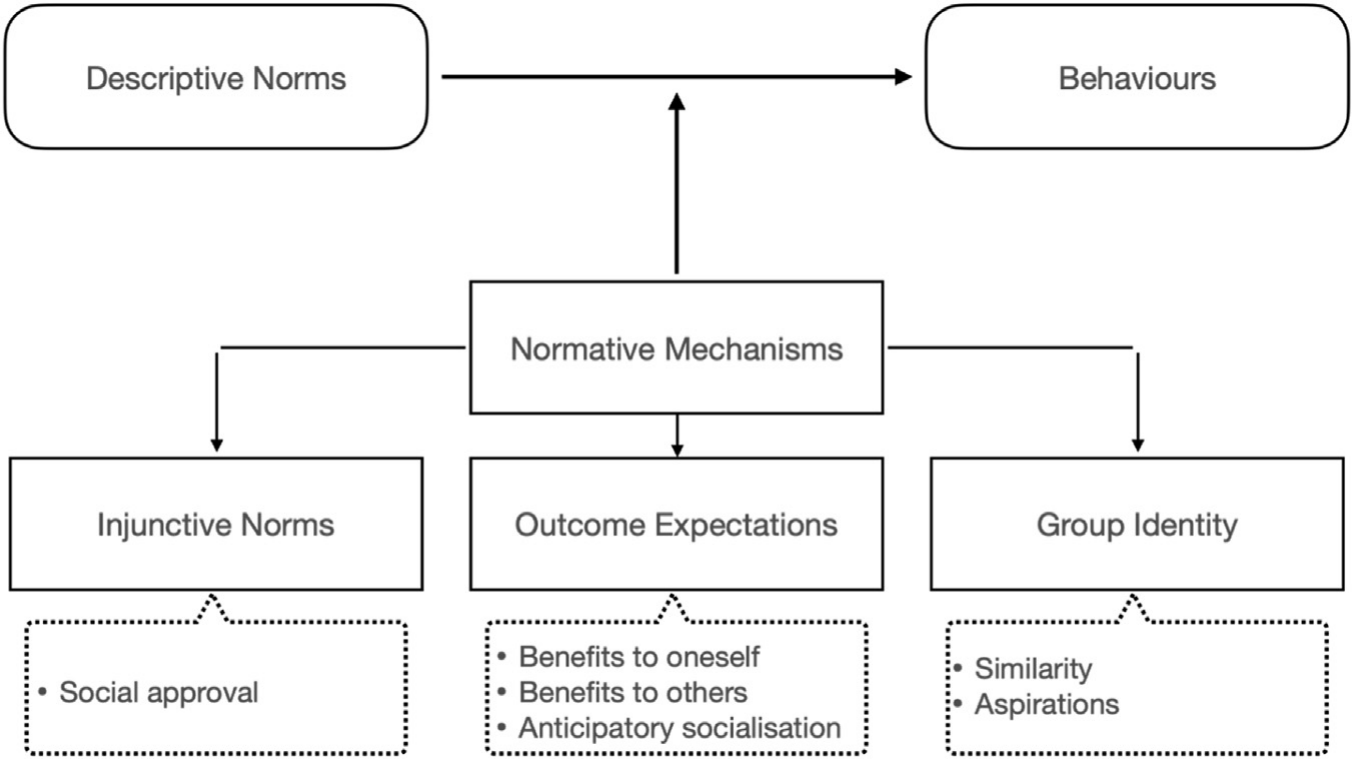
**Components of the theory of normative social behaviour, Rimal et al. (2005).** Colour Cade: Black shapes and grey text, white background.

The Theory of Normative Social Behaviour (TNSB) will be used to elucidate *when*, *how*, and *which* gender related norms affect health behaviours among men and women with PD. The TNSB states that perceived descriptive and injunctive norms may impact behavioural intention, which in turn may impact personal behaviour. Descriptive norms refer to individuals' beliefs about the prevalence of a behaviour (e.g., most women with PD I know, worry about their physical appearance). Injunctive norms refer to the extent to which individuals perceive that influential others or important referents expect them to behave in a certain way and context, and by implication, social sanctions will be incurred if they don’t comply (e.g., women with PD should work on their physical appearance to remain ‘lady-like’).^17^ Hence, as people embody several social roles and identities, it is possible for individuals to believe that certain social peers engage in a behaviour (e.g., people with PD) and simultaneously believe that another groups of social peers (e.g., colleagues) would disapprove of their enacting that behaviour. Therefore, motivations for complying with descriptive gender norms depend on (a) how strong the influence of a certain gender norm is perceived in a particular context (descriptive norms) *and* (b) the importance of the social reference group in an individuals’ embodied identity (injunctive norms). A schematic model of the TNSB is presented in Fig. 2. For this study, we applied the definition of gender norms by Cislaghi et al. (2020), who describe gender norms as ‘*social rules and expectations that define acceptable and appropriate actions, roles and behaviours for women and men in a given group or society*’.^18^ Gender-based stereotypes are ‘*generalised assumptions regarding common traits, roles and behaviours based on a person‘s gender identity or expression*’ and are informed by gender-based expectations.^11^ Whilst gender stereotypes inform our assumptions about another person, gender norms govern the expected and accepted behaviours.

### Sample

In November 2022, self-reported men and women with PD were recruited through the PRIME-NL Gender Study in the Netherlands.^4^^,^^13^ The PRIME-NL Gender Study included a sample of 307 people with PD (127 women and 180 men). People were eligible to participate when they met the following criteria: aged 18 years or older; diagnosed with Parkinson’s disease or Parkinsonism; absence of serious cognitive and/or communication impairment; able and willing to participate in a participatory design workshop of 4.5–5 h (including breaks). Men and women who had previously participated in the PRIME-NL Gender Study and who had indicated an interest to participate in follow-up studies, received an email invitation to participate in the design workshop. In total, 263 participants indicated interest in follow-up studies and received an invitation to participate. The PRIME-NL Gender Study already included a purposive and relatively homogeneous sample of participants with relevant characteristics for this study. Therefore, a convenience sampling method was used with a maximum recruitment goal of N = 40 (20 men and 20 women). For each design session, we intended to include a minimum of 12 participants. Guest et al. (2020) determined that approximately 12 participants would be needed to reach higher levels of saturation in qualitative interviews.^19^

All individuals who indicated to be willing and able to participate in the design workshop received an email confirmation with further information about the workshop objectives, program, and facilitation team. The workshop facilitators (LM, PV, and IG) had no therapeutic relations with the participants and all participants were offered travel reimbursements.

### Procedure and data collection

#### Empathise (Phase 1): understanding gender stereotypes and norms in illness experiences

The aim of this first phase was to understand the role of gender stereotypes and norms in health and illness experiences of men and women with PD and *how* they impact these experiences.

##### Pre-workshop surveys

A week prior to the design session, participants received a pre-workshop survey. The Hoffman Gender Scale was used to assess Gender Self-Confidence (GSC) among participants; the degree to which a person beliefs that they meet their personal standards for femininity/masculinity, which is considered a component of gender identity (Supplement 3).^20^ The survey measures two domains of GSC: Gender Self-Definition, which refers to how salient gender is to one’s individual identity (7-items, e.g., “*When I am asked to describe myself, being female/male is one of the first things I think of*”) and Gender Self-Acceptance, which refers to how comfortable a person feels as a member of their gender category (7-items, e.g., “*I am confident in my femininity/masculinity*”). Each item is scored on a 6-point Likert-scale (1 = strongly disagree to 6 = strongly agree). The mean of all item scores is calculated for each subscale score. The scale has not been validated in a Dutch population; however, this was not deemed a limitation as its function in this study was primarily the establishment of congruent focus groups. Higher scores on the Gender Self-Definition subscale correspond to attributing a greater deal of importance to femininity/masculinity as part of their identity. Higher scores on the Gender Self-Acceptance scale correspond to more acceptance of themselves as female/male without necessarily strongly defining themselves in terms of their notions of femininity/masculinity. The survey is presented in an A (for women) or B (for men) format and includes binary gender congruent statements related to identifying as a woman/being feminine and man/being masculine. The survey contains a final open question which allows participants to define for themselves what the term femininity (for women) or masculinity (for men) means to them. This measure was used as proxy to assess how strongly committed participants were to their gender identity (higher or lower GSC).

The Nijmegen Gender Awareness in Medicine Scale (N-GAMS) was included to assess the degree to which participants are sensitive towards the role of gender in medical care (Supplement 4).^21^ The N-GAMS includes three subscales: 1) gender sensitivity and 2) gender role ideology towards doctors and 3) gender role ideology towards patients. The gender sensitivity subscale (12-items) consisted of attitudinal statements about gender concerns in healthcare (e.g., “*Do you think that addressing differences between men and women creates equity in healthcare?*”). The gender role ideology towards doctors and patients’ subscales assesses the degree to which participants agree with gender stereotypical attitudes towards doctors (7-items, e.g., “*Male physicians put too much emphasis on technical aspects of medicine compared to female physicians*”) and patients (11-items, e.g., “*Women more frequently than men want to discuss problems with physicians that do not belong in the consultation room*”). All items were measured on a 5-point Likert scale (1 = strongly disagree to 5 = strongly agree). This scale was developed and validated in the Netherlands. Notably, the N-GAMS has not previously been applied in patient populations and has mainly been used to measure attitudes among medical students and physicians. However, as the survey includes general statements with regards to gender-sensitive healthcare and generic stereotypical statements in healthcare settings with regards to gender role ideology, the N-GAMS could be considered a valuable instrument to also assess attitudes among patients towards the topic of gender in medical care.

Lastly, with two single item question, participants were asked to respond to the following questions and statement: “*Do you have previous experience with the topic of gender sensitive medicine for men and women with PD, or have you thought about this topic prior to this workshop?*” (Yes/No) and “*I think it is important that there be more differentiation between men and women in PD research and care*” (1 = Strongly disagree, 5 = Strongly agree).

##### Focus group discussions

During the morning program of the design sessions, we conducted focus group discussions (FGDs) using a standardised semi-structured interview guide with open-ended questions. On the first workshop day, we conducted 2 simultaneous FGDs with men with PD and on the second workshop day 2 FGDs with women with PD on the second day. Participants numbers were split equally and allocated to a focus group based on their GSC scores, with one focus group consisting of participants with lower GSC scores and one focus group with higher GSC scores. The objective of the interviews was to explore and identify gender norms or stereotypes that impact illness experiences of men and women with Parkinson’s Disease. We hypothesised that gender norms or stereotypes would be more salient in the experiences of participants with higher GSC scores compared to those with lower GSC scores. IG, LM and PV developed the interview guide, which was discussed with SOP (Supplement 5). The FGDs were performed simultaneously by LM and PV and observed by IG. The interviews were audio-taped and lasted 75–90 min.

#### Define (Phase 2): individual focal points for gender-aware PD care

Participants were individually guided through 3 self-reflective design-based methods to evoke individual insights towards relevant objectives for gender-aware PD care: 1) Reverse Thinking, 2) Word-Concept Association and 3) Download the Learnings.^22^ The purpose of Reverse Thinking was to prompt ideas about ‘*how gender-sensitive care for people with PD would look like if we designed it completely wrong*’. The Word-Concept Association was meant to elicit individual associations with gender-specific care for men or women with PD and the Download the Learnings exercise was intended to create a personal description for gender-sensitive PD care and when this becomes most relevant for the individual participant. The duration of this phase lasted 20–25 min.

#### Ideate (Phase 3): formulate key recommendations for gender-sensitive PD care research

The objective of this final design phase was to cumulate the learnings of phase 1 and 2 to formulate recommendations for gender-sensitive PD care. In subgroups of 3–4 people, participants shared their learnings from Phase 2 and were encouraged to listen for common themes and discuss differences. Subsequently, collective insight statements were created, answering the following prompt: “*For us, care for men/women with Parkinson means paying attention to [……] This is especially important when [……]*”. Lastly, each group was asked to reflect on recommendation for future research into gender-sensitive care for men and women with PD. Each subgroup was invited to present their collective insights during the plenary closing of the workshop. The duration of this phase lasted 60–75 min.

#### Notice and reflect (Phase 4): research team and reflexivity

The notice and reflect phases are ongoing and throughout the design and research process. In this study, we operationalised these phases through reflective discussions prior to- and directly after the workshop among the facilitation team (IG, LM, PV). The intention of these phases is to encourage self-awareness and introspection among the facilitation team members about potential biases and assumptions that can influence the research process. For example, the facilitation team was aware that it consisted of 3 female team members and facilitated both the women’s design session and the men’s design session. Furthermore, the preliminary workshop program was discussed with SKLD, neurologist-in-training, to ensure a workshop program, methods and location that were accommodating towards the potential cognitive and physical challenges of people with PD. As a result, we introduced self-reflective exercises in phase 2 of the workshop and included frequent breaks to lower the stress that can be triggering through prolonged cognitive- and interactive group work for people with PD. To accommodate participants with PD related handwriting challenges, we opted for larger papers and digital typing options instead of the typical ‘post-it notes’, which are often used in design sessions. After the workshop, the facilitation team completed an online evaluation reflecting on the workshop program, process and discussed the outcomes with specific focus on principles of inclusive and equity centred design.^23^ The evaluation questions can be found in Supplement 6. These types of reflections are an integral part of the equity-centred design process and of the ‘*notice and reflect*’ phase in particular.

### Data analysis

#### Statistical analysis

##### Pre-workshop surveys

Descriptive statistics were performed on the demographic data of the participants, the Hoffman Gender Scale, and the Nijmegen Gender Awareness in Medicine Scale. Differences between men and women in demographic data and the survey scale scores were compared using the Kruskal–Wallis rank sum test or the Fisher exact test. Missing responses were removed during analyses. Data was analysed with the use of R Studio (version 4.1.3).

#### Qualitative analysis

##### Phase 1: focus group interviews

The interviews were audio-recorded and professionally transcribed ad verbatim. To explore which gender stereotypes and gender norms are made relevant by participants, a reflexive thematic analysis was applied to the two FGDs with men and the two FGDs with women separately.^24^ Themes were compared for within-group similarities and differences between the higher and lower GSC subgroups regarding experiences with gender norms and stereotypes in their illness experiences. Additionally, we applied the TNSB to analyse at which level of social manifestation gender norms and stereotypes were experienced by men and women with PD. For this, the social manifestation levels as described by the equity-centred design guide were used: 1) ideological level (descriptive norms), 2) interpersonal level (descriptive/injunctive norms) and 3) internalised level (injunctive/self-stereotyping norms).^23^ The analyses were guided by the following questions: “*Which gender norms or stereotypes are present in the illness experiences of people with PD?*” and “*At which level(s) of systemic social structures do these gender norms or stereotypes occur?*”. First, the text was read and re-read to familiarise the researchers with the data. Secondly, open coding was applied by LM and IG to identify meaningful text units related to the research question. Data was coded with the use of ATLAS.ti (version 22.0.11). In this stage, the text that explicitly addressed gender norms or stereotypes in illness experiences were separated from the text for further analyses. Codes were discussed and categorised according to similarities and differences in content. An iterative process of identifying and defining gender norms and stereotypes was performed. Lastly, LM and IG analysed and labelled each gender norm and stereotype on the level(s) of social manifestation in which they were mentioned by participants.

##### Phase 2: Self-reflective methods

For this paper, the results from the Word-Concept Association (WCA) method and the Downloaded learning are presented. The self-reflective prompting exercises were recorded in written notes. The WCA methods was translated with the use of Deepl.com and visualised using Word Clouds, generated by ATLAS.ti. Word Clouds are a graphical ranking system, i.e., the more a word is mentioned by the participants, the larger it will be visualised in the Word Cloud. The data from the Downloaded Learnings methods were descriptively analysed. An overview of the self-reflective methods can be found in Supplement 7. The Reverse Thinking method was translated with the use of Deepl.com and the results can be found in Supplement 9.

##### Phase 3: collective insight statements and care research recommendations

The collective insight statements and research recommendations were video recorded during the plenary presentations, recorded in written notes and descriptively analysed. Preliminary findings of the focus group interviews, and a summary of the collective Phase 3 outcomes were shared with participants one week after the participatory design sessions to provide an opportunity for participant feedback and reflections on the workshop.

### Role of the funding source

All co-authors had access to the data in the study, and they accept responsibility to submit for publication. The funding sources were not involved in the study design; in the collection, analysis, and interpretation of data; in the writing of the report; and in the decision to submit the paper for publication.

## Results

### Participant characteristics

Overall, 21 men with PD and 18 women with PD registered for the workshops, of which eight men and three women cancelled their participation before or on the day of the workshop. Reasons for cancellations were related to not feeling well enough to travel, not having enough energy to join the workshop or the emergence of other commitments. In total, 28 people participated in the two workshops: one workshop with 13 men and one with 15 women. All participants’ sex assigned at birth was congruent with their gender identity, leading to a study cohort of only cisgender individuals. The median (IQR) age of the participants was 64.2 (7.8) years, and all participants were of Dutch descent. Most participants lived together with a partner or family (96%) and 29% had paid employment. Participants had a median disease duration of 4.9 (2.4) years; notably, with women having a significantly longer median duration (5.9 (2.7) years) compared to men (3.7 (1.4) years) (p = 0.016). Table 1 shows the demographical and clinical characteristics of the study population.

**Table 1:** Demographical and clinical characteristics of the participants.

Characteristic	Overall (N = 28)	Men (n = 13)	Women (n = 15)
**Age** (Median in years (IQR))	64.2 (7.8)	64.9 (6.5)	63.5 (8.9)
**Disease duration** (Median in years (IQR))	4.9 (2.4)	3.7 (1.4)	5.9 (2.7)
**Hoehn & Yahr Score**^24^ (stage) (n(%))			
Unilateral involvement only (1)	15 (54)	7 (54)	8 (53)
Mild bilateral involvement (2)	8 (29)	4 (31)	4 (27)
Moderate bilateral disease (3)	4 (14)	2 (15)	2 (13)
Severe disability, but still able to stand and walk (4)	0 (0)	0 (0)	0 (0)
Wheelchair bound (5)	1 (3.6)	0 (0)	1 (6.7)
**Ethnicity** (n(%))			
Dutch	28 (100)	13 (100)	15 (100)
**Education level**^a^ (n(%))			
Lower education	2 (7.1)	1 (7.7)	1 (6.7)
Medium education	6 (21)	3 (23)	3 (20)
Higher education	20 (71)	9 (69)	11 (73)
**Living situation** (n(%))			
Alone	1 (3.6)	0 (0)	1 (6.7)
With partner or family	27 (96)	13 (100)	14 (93)
Facilitated care	0 (0)	0 (0)	0 (0)
**Working status** (n(%))			
Working	8 (29)	3 (23)	5 (33)
Not working	20 (71)	10 (77)	10 (67)

a For education levels, the following categorization is used: Lower education (None, Primary education, VMBO, MAVO), Medium education (HAVO, VWO, MBO), Higher education (HBO, University, PhD).

### Pre-workshop survey results

Results of the two single item questions showed that most participants had no previous knowledge or experiences with the topic of ‘*gender sensitive care for men and women with PD*’ (85%) and had a slightly positive attitude towards the importance of gender sensitivity in PD care (3.8 (0.9) on a 1–5 range). The mean (SD) Gender Self Confidence score of the participants was 4.1 ((1.1) on a 1–6 range), indicating that the degree to which participants met their own standards for masculinity (for men) and femininity (for women) was moderately strong. Participants’ definitions of masculinity or femininity can be found in Supplement 8. Furthermore, participants considered their masculinity or femininity as a moderately strong component of their overall identity (3.9 (1.3) on a 1–6 range). The results of the N-GAMS showed that the attitude of the participants towards gender-related concerns in healthcare was slightly positive (3.8 (0.5) on a 1–5 range), with women having a more positive attitude towards gender-related concern in healthcare (4.1 (0.5)) compared to men (3.5 (0.4)). Furthermore, participants had a neutral attitude about gender stereotypes towards doctors (2.7 (0.5) on a 1–5 range) and slightly disagreed with gender stereotypes towards patients (2.3 (0.4) on a 1–5 range), with men having a more neutral Gender Role Ideology towards patients (2.5 (0.4)) compared to women (2.2 (0.4)). Two women did not complete the single items question regarding previous knowledge or experiences with the topic and one woman did not complete the N-GAMS. Their responses are not included in pre-workshop survey results presented in Table 2.

**Table 2:** Pre-workshop survey results of the participants.

Domain	Overall (N = 28)	Men (n = 13)	Women (n = 15)
Previous topic knowledge or experience (No) (n (%))	22 (85)	12 (92)	10 (77)
Importance of gender sensitivity in PD score (Mean (SD))	3.8 (0.9)	3.7 (0.8)	3.9 (1.0)
Hoffmans’ Gender Self-Confidence score (Mean (SD))	4.1 (1.1)	3.9 (0.8)	4.2 (1.3)
Gender self-definition	3.9 (1.3)	3.7 (1.0)	4.0 (1.5)
Gender self-acceptance	4.3 (1.1)	4.2 (0.8)	4.4 (1.2)
N-GAMS gender sensitivity score ((Mean (SD)^a^	3.8 (0.5)	3.5 (0.4)	4.1 (0.5)
N-GAMS gender role ideology towards doctors score (Mean (SD))	2.7 (0.5)	2.7 (0.4)	2.6 (0.6)
N-GAMS gender role ideology towards patients score (Mean (SD))^a^	2.3 (0.4)	2.5 (0.4)	2.2 (0.4)

a p < 0.05 indicates statistical significance.

### Phase 1: focus group discussion results

Several gender related stereotypes were expressed by the participants on a different level of social manifestation. Overall, the most prominent gender-related stereotype mentioned by both men and women with PD was the idea related to “*People with PD are old men*”. This stereotypical idea is disease (‘Parkinson’s’), age (‘old’) and gender identity (‘men’) specific and often impacts the prospects of living with the disease and affects the experiences of stigma in people of different ages and particularly women. Some men and women also expressed stereotypical ideas related to dealing with difficulties in daily life, where coping through excessive drinking is considered more acceptable for men, whereas compulsive shopping was more acceptable for women. We found no distinct differences in experiences with gender norms or stereotypes between the higher GSC groups and the lower GSC groups, indicating that these themes are not necessarily related to how strongly participants identified with- and were committed to their masculinity or femininity. Table 3 provides an overview of the gender stereotypes and norms mentioned by the participants, in order of prevalence, reflecting the relative saturation level of each item across the focus groups.

**Table 3:** Gender stereotypes in illness experiences of men and women with PD.

Gender stereotype or norm	Expressed by men/women	Level(s) of social manifestation	Exemplar quotes
People with PD are old men	Men and women	Ideological/Interpersonal	“*I couldn't identify well with the diagnosis at all,[…] I saw an eighty-year-old man who was walking with difficulty. So, I thought, how can I have it? And that still causes a little bit of yes, not being able to identify with the fact that I also have it. And that it is completely normal for women to get it too, of course.*” [Woman] “*When they said to me ‘you have Parkinson's’, then you already have an image that is this old, deficient man who walks all bent over and indeed can't do anything anymore and has to sit in a chair. That's ultimately, that's kind of your vision of the future.*” [Man] “*It is also because it is confirmed over and over again. Other people, including girlfriends, say to you "hey, how can that be, that's an old man's disease. And well, then you have to explain it, and it's actually very strange to have to explain it. So yeah, then I feel like I'm one zero behind or something and then I get the feeling it's not about me.*” [Woman] “*When I tell someone for the first time, they say, but you're not that old yet. I do think that's a stereotypical comment. That it's really an old age disease and that you're already....* *SP1*^a^*So you must be an older man?* *SPR*^a^*Yes.* *SPR Who shakes a lot*” [Men]
Men should suppress their emotions	Men	Internalize/Interpersonal	“*There might be something masculine about that too, if we’re talking about gender, which we men, we’re not ...* *SPR We don’t cry as easily no.* *SPR Finding that more uncomfortable than maybe a woman or something.*” [Men] “*I have a female specialist. Well, she knows how to get me to shed a tear [...] But then she continues to ask me questions like that and then I get embarrassed, tears come like that. But would that [specialist] be man, I think, I’d be even more embarrassed.* *SPR Or would he not ask those question?* *SPR Asking those same question.* *SPR No, or would that man, that male doctor not have asked that question?*” [Men]
Men should be strong	Men	Internalized	“*I was always a pretty confident person and I’m a lot less so now. And also, when you see yourself move sometimes in the mirror, you think, you are so crooked, you know. That shows a lot less masculinity than it used to. And I sometimes have trouble with that.*” [Man] *But what I notice is that I have trouble that my masculinity, we can talk about that in a moment, what that means exactly, that’s being tinkered with, that’s being nagged at, so to speak. So, performing in mountain biking, performing in all kinds of other sports yes, you notice that is going to be less. And I find that very difficult. The feeling of being a man and wanting to perform in being strong, being powerful.* [Man]
Men are the providers	Men	Ideological/Interpersonal/Internalized	“*All my life, I was raised as a Marine, a professional Marine, from a Reformed family. So that’s a stamp on your worldview. And yes, I was brought up that as a man that you have things taken care of, that the household has adequate resources to run. And my father had ten slogans, one of them was you can’t be afraid, you have to take the bull by the horns. And you try to do that. And then morally you get something and that’s going to hinder you. Then you become dependent on your wife, I have a wonderful wife it’s not about that. But I don’t want to be dependent on her. I say you're married to me as a guy, but not ’s a caregiver. And I find that very annoying.*” [Man] “*And I think we are all still from the generation when there is a fairly old-fashioned male-female hierarchy in the home anyway. One does this and the other does that. But with us too, it’s going to change. My wife is also quite old-fashioned, so she keeps pushing everything off, like you arrange it. So that will be quite a challenge in future.*” [Man]
Men with addictions drink more, women go shopping	Men and women	Ideological	“*I fortunately don’t have a problem with alcohol myself, but I do know of the necessary men who indeed if they have a problem then they drink more.* *SPI Yes, is expressions of addiction you say there is something male about that?* *SPR Well, addiction not so much, but maybe the manifestation. The stereotype is that we grab the alcohol more and a woman goes to the store more so to speak.*” [Man] “*Yes, the disinhibition in men is more likely to be in sex and in women somewhere else, in expressing feelings, or not expressing them at all, or a little bit snarky.* *SPR Disinhibition is just a little bit different?* *SPR Yes.* *SPR Yes, the expression is different.*” [Women]
Women are the family carers	Women	Ideological/Interpersonal/Internalized	“*What I also notice with couples with Parkinson’s, everyone thinks it’s very normal when a woman takes care of a man with Parkinson’s. But that they find it very special when a man takes care of his wife with Parkinson’s, then suddenly that is a hero. For that woman, that’s normal.*” [Woman] “*But the husband was sick and then he could also immediately do almost nothing. But when a woman is ill, you stay at home and take care of things. And then they are told by the doctor ‘well it won’t be so bad, because she is still hanging up the laundry’. While you say you can’t move your arm.* *SPR You should see how I hang the laundry, you know like that.* *SPR So what else do you have to say.* *SPR So you have to conceal things sometimes too.* *SPR Yes that won’t be too bad.* *SPR Yeah right.* *SPR Can you still run your household? I said of man you don’t want to know how I do it, but you do it.* *SPR You can hardly say no because indeed you do.* *SPR Then it must be not too bad.*” [Women]
Women should look friendly	Women	Ideological	“*Women are supposed to look a little more friendly.* *SPR And they do feeling supporting gestures and with their face too. And if you don‘t do that then they think you‘re uninterested.* *SPR Yes, because you’re such a dragon, such a bitch or such a nasty woman.* *SPR And with a man that doesn’t happen, or he was drunk, but not that he’s a nasty man.* *SPR I have to think about Prince Claus, of course that was someone you saw a lot in public. That wasn’t one of those stereotypes about I thought ‘hey, what an unsympathetic man’. That was more of ‘oh my, you can tell by looking at him that he has Parkinson’s.’* *SPR That’s kind of accepted that’s Parkinson’s, but with the women it takes longer for that understanding of it. Initially she’s cranky.*” [Women]
Women physicians should be emphatic	Women	Ideological/Interpersonal	“*I notice with doctors and health care providers; some are more empathetic than others. And when I tell of the experience with two neurologists, they think the empathetic doctor was the woman and the non-empathetic doctor was the man. But it was exactly the other way around. So the stereotypes are there too ...* *I also think maybe as a woman you appreciate more if a man is empathetic to you and a woman you expect it maybe more. Yes, I don’t know, if I look at myself then yes, I do appreciate that yes.* *SPR Then if that woman as a doctor is not empathetic, I think it’s almost worse.* *SPR That’s what you say too, yes that you actually expect it.* *SPR I find that so bad that I don’t go there anymore.*” [Women]
Men should be good sexual partners	Men	Ideological	“*But I think then, that’s also something I just heard, performing, it also belongs to ‘sexual performing’ in quotation marks. Not that I experience it that way, but that is also something that is very important for men often and for men in general. That you are good at sex so to speak.*” [Man]

a SPI = interviewer, SPR = respondent(s).

Among the men with PD, there was an internalised sense of gendered stereotypes related to “*Men should be strong*”. Men should have the ability to be independent, be physically and mentally strong and perform well in sports and sexually. PD motor- and nonmotor symptoms impact these abilities, resulting in feelings of loss of control, confidence, and independence. At multiple levels, men expressed a general idea related to “*Men should suppress their emotions*”, describing situations in which men should control their emotions, particularly in the context of crying. Men expressed the importance of asking (follow-up) questions because they often felt less inclined to share emotionally loaded experiences due to discomfort and unfamiliarity. This need is dependent on personal openness and willingness to share and experienced level of social support. The idea of “*men are the providers*” was related to men’s perceived responsibility as providers for their family and not becoming dependent on their (female) partner.

Among the women with PD, the gendered stereotype of “*Women are the family carers*” was related to the normalisation of women being the household- and informal caretakers. Women indicated that the performance of these roles can mask difficulties with PD-related symptoms observed by others due to their continued participation in these activities and social roles. Women described themselves as adaptive by having various compensation strategies to juggle the challenge of performing multiple social roles whereas it was generally viewed that men would ask for- and receive help sooner. The belief that “*Women should look friendly*” was related to how changes in body language, posture, and facial expressions due to PD symptoms resulted in stigmatising experiences for some women, as women are often socially expected to be more emotionally and nonverbally expressive. One group of women also expressed the stereotypical idea that “*women physicians should be empathic*” and that women physicians might face stronger social criticism, also by their own gender peers, when they are not considered ‘empathic enough’.

### Phase 2: self-reflective methods

#### Word-concept associations

**Fig. 3:**
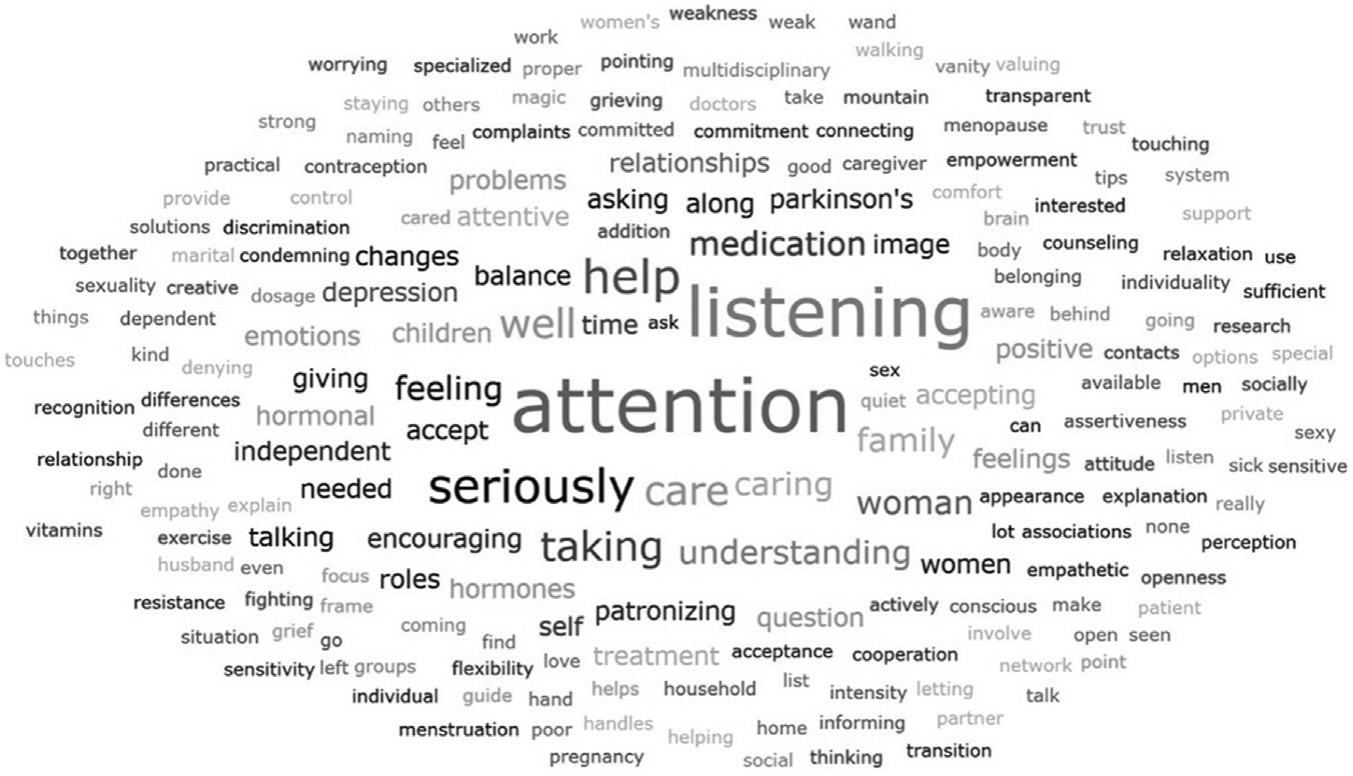
**Word-concept association: care for women with Parkinson’s disease.** Colour Cade: Black, grey, white background.

The findings of the word-concept association show that women relate care for women with PD with interpersonal traits such being listened to and taken seriously with attention towards balancing the different social roles’ women fulfil in their lives. Clinical associations focused on attention towards the impact of hormonal changes and differences in medication dosage and treatment responses (Fig. 3).

**Fig. 4:**
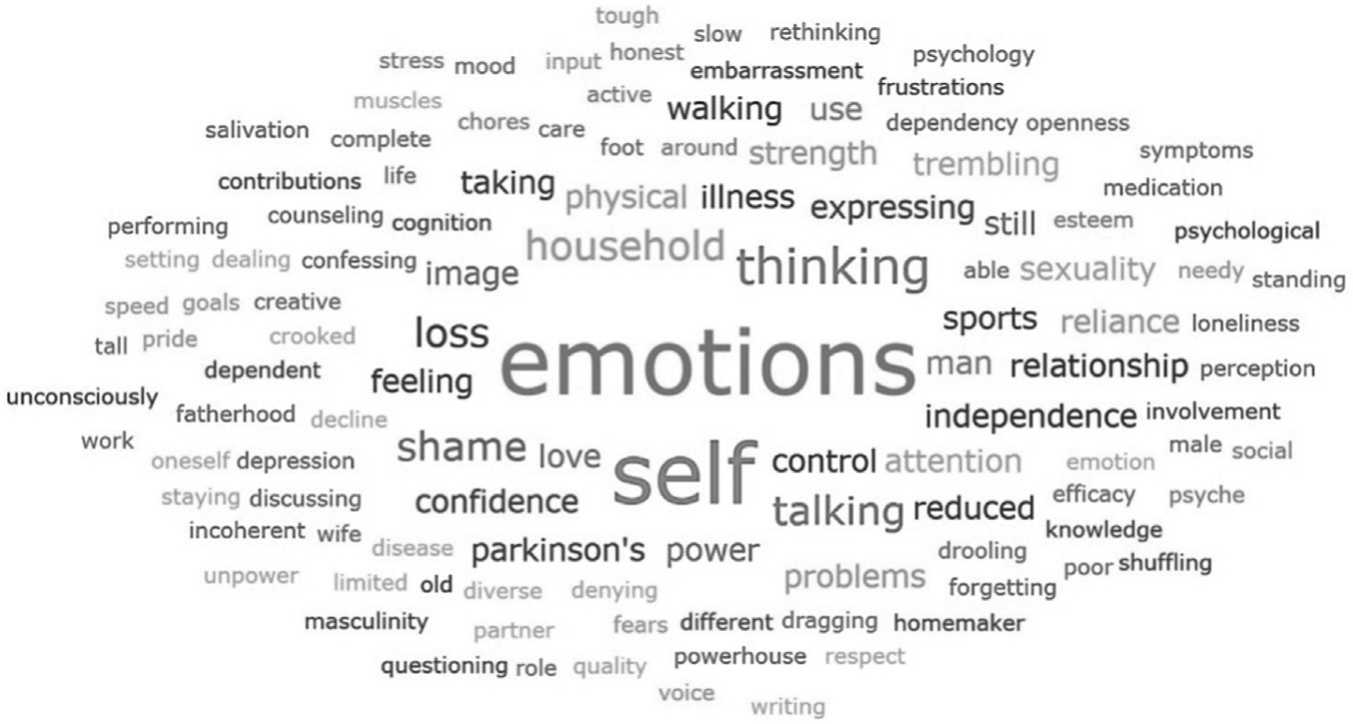
**Word-concept association: care for men with Parkinson’s disease.** Colour Cade: Black, grey, white background.

For men, the word-concept associations present attention towards dealing with changes and difficulties in expressing emotions, and a focus towards self-management and self-reliance. Also, associations were made with men’s roles as husbands and fathers, feelings of shame and loss of confidence and physical abilities (Fig. 4).

#### Downloaded learnings

Results of the Downloaded Learnings exercise showed a variety of focal points for gender-aware PD care as described by the participants. Both men and women described a, more sex-linked, focus on potential differences in side effects of PD medication and optimal dosage for men and women. An attention towards personality and life changes and dealing with- and listening to the emotional experience of living with PD were also prominent topics for both genders. Men described a difficulty between balancing a ‘do not complain and carry your emotions’ attitude while also feeling relieved when they were able to express their emotional experiences. Women mentioned a call towards challenging the stereotypical image of ‘the person with Parkinson’ due to its strong gendered association with ‘an old man’s disease’ and the need to move beyond the stereotype for a more accurate representation of the diversity of people who have the disease.

### Phase 3: collective insight statements and care research recommendations

Results of the small group discussion among men revealed an eminent focus of gender-sensitive PD care for the psychosocial experiences of men with PD related to their self-image, self-confidence, and self-reliance. This includes attention to personality and cognitive changes. Men stressed the relatively strong focus on monitoring and treating the physical aspects of Parkinson’s but the lack of attention to emotional well-being, particularly in the period after diagnosis, and coping with increased emotionality and mood disturbances. Additionally, changes in sexual experiences, both physically and emotionally, require more attention. Preferably these topics are discussed initially in their direct or relevant social environment with partners and close friends, but men stated the importance to address these topics in clinical practice as well.

The small group discussions among women identified a need for gender-sensitive PD care with more attention towards emotional well-being of women with PD and a focus on (re)enforcing self-agency and equivalence in the patient-physician relationship. Women stressed the importance of attention to women in different life stages (related to age) and life situations (e.g., active (single) parenting role, caregiver for partner, working professional) that might require different levels of flexibility in care demand and supply. Furthermore, the women expressed the need for more knowledge regarding the impact of hormonal influences on PD progression and treatment. An overview of the formulated care and research recommendations for future gender-sensitive care research for people with PD is included below (Table 4).

**Table 4:** Gender-sensitive PD care and research recommendations.

	Care & research recommendations
1.	Create more balance between physiological and psychosocial skills and expertise within multidisciplinary care teams for people with PD.
2.	Investigations into emotional- experiences, changes, challenges and growth among men and women with PD.
3.	Investigation into care needs among men and women with PD in different life stages and circumstances (life-course perspectives).
4.	Create more awareness regarding gender stereotypes and prejudices when formulating research questions and analysing research data, to avoid reproduction of restrictive gender biases.
5.	Continued investigation into men-specific and women-specific issues by more explicitly exploring and addressing them during medical consultations and research.
6.	Proactively discuss social role changes with men and women that are affected by the progression of PD symptoms.
7.	Continued efforts for structural research into similarities and differences in biological sex characteristics and social-cultural gender dimensions in diagnostics, treatment, therapy and care for men and women with PD.

## Discussion

In this multi-method design-based study, we explored patient perspectives on gender norms and gender-sensitive care. Most participants had no previous knowledge or experience with the topic of ‘*gender sensitive PD care*’ and had a positive attitude towards investigating and addressing gender related concerns in healthcare. Participants considered their masculinity (for men) and femininity (for women) as a moderately strong component of their identity and overall, participants did not hold strong gender stereotypical attitudes towards male and female patients and physicians. Furthermore, we found that there are shared and distinct gendered stereotypes and norms that influence illness experiences of men and women with PD. Most prominently, the general perception that ‘*People with PD are old men*’ impacts both men and women’s own initial perception of living with the disease and the perception of their social environment. Attention towards personality changes, dealing with- and listening to emotional experiences of living with PD were prominent topics for both men and women, although their interpretations differed. Men described their experiences with prevalent gendered stereotypes related to ‘*men should be strong*’, ‘*men should suppress their emotions*’ and ‘*men are providers*’. Women described experiences with stereotypes related to ‘*women are family carers*’ and ‘*women should look friendly*’.

The results from the self-reflections revealed that women associated ‘*gender-sensitive care for women with PD*’ with a focus on interpersonal aspects related to the patient-healthcare provider relationships such as being listened to and attention towards the different social roles women fulfil in their lives. Men tended to associate ‘*gender-sensitive care for men with PD*’ more with attention towards intrapersonal aspects such as self-regulation, management, and reliance. Both men and women expressed a need for more attention towards emotional wellbeing. This is particularly important in the period after diagnosis when people with PD are learning to cope with increased emotionality due to PD symptoms and disease progression and the impact of PD treatment on social role changes. Participants expressed a need for emotional support with a focus on (re)enforcing self-agency.

Recommendations for gender-sensitive PD care and research included fostering awareness among researchers and healthcare providers about gender stereotypes to avoid the reproduction of gender biases as well as the encouragement to proactively address social role changes due to the progression of PD related symptoms. For healthcare providers, this begins with acknowledging the emotional toll that social role changes can have on patients and understanding feelings of frustration, loss and anxiety related to changes in the ability to perform certain roles. This includes reflection on your own, perhaps unconscious, assumptions regarding gender norms and roles and how these might influence (medical) social interactions. Also, it is important to assess the specific challenges in peoples’ personal and professional roles to tailor their individual treatment plans and provide education about how disease progression can lead to changes in motor functions, energy levels, emotionality, and cognitive abilities. This can contribute to an understanding that these changes are a result of underlying neurodegenerative processes and not personal failing.^25^ Furthermore, discussing adaptive strategies, assistive technologies and mobile aids that can help people with PD to continue to engage in their desired social roles with modifications in behaviours, routines, and environments. Researchers are encouraged to integrate gender-sensitivity training and collaborations with advocacy groups focused on gender equity in healthcare to enhance the recognition of gender stereotypes and understanding of the impact of undesirable social biases on patient outcomes.

The stereotype of ‘*People with PD are old men*’, while well supported, is the most common representation of PD since the sketch by Sir. William Richard Gowers MD in 1886.^26^ Although this image is still accurate for some people with PD, it does not represent the vast variations among people with PD we know today. As a result, particularly younger and middle-aged women can face poor public understanding and experience an unnecessary explanatory burden to counter this stereotypical perception.^6^ We therefore echo the calls for a broader and more accurate view of Parkinson’s through the use of diverse imaging and inclusive research participation.^27^, ^28^, ^29^, ^30^ While acknowledging that no single image can adequately reflect the diversity in background, phenotypes, and experiences in PD, it is important that images, both in public and in medical teachings, are consistent with the advances in Parkinson’s research and encourage discussion about how Parkinson’s is represented.

In this study we found gender norms and stereotypes on multiple levels of social manifestation in illness experiences of men and women with PD. Among men, the gender norms related to ‘*men should be strong*’ and ‘*men should suppress their emotions*’, were most strongly internalised and are therefore more likely to act as prosocial self-regulators. Research suggests that, although men and women tend to have similar emotional reactivity and fluctuations, men are more likely than women to suppress emotional expression in certain situations.^31^^,^^32^ A recent study reported greater gender role flexibility in women compared to men, which refers to the capacity to contextually switch between self-perceived masculine and feminine behaviours. Men experienced more negative affectivity, such as increased anxiety, self-criticism and feelings of depression when ‘code switching’ gender roles.^33^ The collective insight statement of men in our study that calls for more psychosocial support and coping with changes in their self-image, self-confidence, and self-reliance require care approaches that are sensitive towards these gender normative constrains regarding the expression of emotional experiences. Particularly those that are considered stereotypically feminine and gender role inconsistent for men (e.g., fear, sadness, embarrassment, shame or guilt).^34^ It is well known that maladaptive emotional regulation increases psychological stress, exacerbates motor symptoms and result in poorer health outcomes in PD.^35^ Especially for men who consider their masculinity a strong component of their overall identity and define their masculinity in traditional, hegemonic, terms, emotional disclosure can be challenging. These restrictive masculine norms and self-stereotyping behaviours are illustrative of a gendered pathways to health that can limit men’s access to and utilisation of psychosocial PD care.

It is important to note that masculinities demonstrate a wide range in patterns of practice and a generalisation about emotionality and men would be misleading. Focusing on masculinity risks being overly focused on problems associated with ‘negative’ masculinity and may neglect to focus on adaptive traits and the normalisation of emotional experiences.^36^ Gender transformative approaches to improve emotional health and wellbeing of men with PD require not only changes in personal narratives but also in media representations, healthcare discourses and care services that normalise mental health, integrate role modelling and leverage adaptive gender norms and value systems.^18^^,^^37^ This is particularly relevant in a strong national gender equality discourse in which women’s emancipation promotes non-traditional role changes for women and dynamic feminine stereotypes, whereas the transgression of men into social roles that are generally considered communal or feminine progresses much slower and traditional masculine norms seem much more resistant to change.^38^, ^39^, ^40^ It is important for researchers, clinician and policy-makers in healthcare to recognise that cultivating dynamic and less restrictive masculinities, normalising mental health and acknowledging the diverse ways in which men may construct gender ideals, *is* psychosocial health promotion for men with, and without, PD.^10^^,^^36^^,^^41^^,^^42^

Gender norms related to ‘*women as family carers*’ were most strongly internalised among the women in our study. Norms related to ‘*women should look friendly*’ were largely experienced by women as ideological, descriptive norms. A recent study into emotional cues in expressive behaviours of men and women with PD found that, to conform to gendered social expectations, women with PD may experience more pressure to express sociable behaviours such as more smiling and laughing during conversations. This study suggested that these socially desired behaviours can be misunderstood by observers, even healthcare professionals, and mask negative emotional experiences of women with PD, particularly when smiling and laughing is expressed with less conversational engagement.^43^ These findings are in line with the remarks of women in our study that the performance of certain social roles, such as active family care and household duties, can mask experienced difficulties with PD-related symptoms observed by others. Subsequently, this can lead to inaccurate evaluations of emotional and physical well-being of women with PD, intensify feelings of not being listened to or taken seriously and could explain why the women in this study described their need for- and use of more adaptive coping strategies compared to men. These examples are illustrations of a gendered pathway to health that can hamper women’s access to care and therefore increased awareness of these gendered performances and potential biases is needed among healthcare professionals. This could be assisted with active listening skills and additional probing during medical consultations to assess emotional and physical experiences of women with PD in a more detailed way.^44^ We support the ‘call to arms’ described by Subramanian et al. (2022) that management of PD needs to be customised to include the unique stages and roles of women’s lifes.^45^

A strength of this study is the in-depth line of inquiry through our multimethod approach. For this present study, we applied an equity-centred design (ECD) approach that incorporates intentional reflectivity and that acknowledges power, identity and context in which the design process takes place.^14^^,^^23^ This multi-method participatory process includes patients in the research and design process and aims towards a practical translation of generated knowledge and insights. In a collaborative process, people with PD and health researchers worked together using cultural inquiry to understand patients lived gendered experiences, creative thinking to stimulate diverse perspectives and prioritise ideas related to gender aware PD care. It is useful to note that, in contrast to more traditional hypothesis-driven biomedical research, the design-based approach we employed in this study centres on understanding complex real-world contexts, aiming not to prove or reject hypotheses, but to comprehensively explore multifaceted issues and generate patient-driven recommendations through an iterative process of problem identification and solution co-creation.

To our knowledge, there are still few design-based health research studies that directly address gender inequities that impact health and illness experiences.^46^, ^47^, ^48^, ^49^ While human-centred design methodologies, such as the ECD approach used in this study, are often perceived as a single standardised method, their application, in fact, entails a wide array of qualitative, quantitative and design methods or techniques that can and should be used selectively, dependent on the specific research context.^50^ ECD practitioners in health research should carefully select the participatory methods they deploy, with sensitivity towards capturing the unique insights and capabilities of each participant in the design process and stay attuned to the power dynamics and agency of varying stakeholders during co-design sessions. Using an ECD approach can support the development of care interventions that increase gender equity in PD care. When men, women and gender diverse people with PD are encouraged to use the power of their own lived experiences to identify and discuss gender-related experiences and ideate priorities for gender-sensitive PD care, community members are purposefully involved as co-designers with the goal of formulating and innovating solutions relevant to their needs. This intentional engagement of patient communities is especially important when it comes to improving the knowledge base around PD, which is still based largely on the experiences of a relatively homogeneous population from European Caucasian descent.^28^^,^^51^ To foster genuinely inclusive design research processes, using reflective tools and frameworks, such as ECD, can ensure that a participatory process is not just tokenistic but genuinely meaningful.

It should be noted that the sample in this study is not necessarily representative of the broader Parkinson’s population in the Netherlands. Participants had generally received higher education, experienced mild to moderate disease disability and were not as ethnically diverse as the general Dutch population. Homogeneous sampling is employed when the objective is an in-depth exploration of the experiences of a particular group by minimising group differences; this approach could therefore be viewed as a strength for this study. Nonetheless, there are increasing demands to emphasise greater diversity and inclusion in Parkinson’s research overall.^28^ It remains to be investigated whether the same pattern of results appears when studies are conducted that include individuals with more advanced stages of disease progression and with distinct social identities that might adhere to- and practice different gender norms and stereotypes.^6^ The promotion of an intersectional gender approach that emphasises the intersectionality of gender with other contextual aspects of identity, such as ethnicity, socioeconomic status, sexual orientation, and age, is needed to better understand and address the unique healthcare challenges faced by men and women with PD from different social backgrounds.

Furthermore, investigations could be even more robust if researchers considered the added layer of complexity brought by disease-specific symptoms that intensify during the disease journey. For example, researchers could consider how contextual gender norms related to caregiving can become more or less salient with disease progression and shape the quality of life of both men and women with PD and their caregivers.^52^ Or when one considers that depression, common in Parkinson's disease, combined with its potential gendered manifestations can play an important role in help-seeking behaviours and may hold particular significance for people with PD that endorse traditional masculine gender norms.^53^^,^^54^ Such intricate interplays necessitate more nuanced research approaches, that can shed light on the compounded influences of social identity, gender norms, and disease-specific symptomatology. Herein, contextualisation should be prioritised over broad generalisation, as these considerations steer away from a one-size-fits-all paradigm and towards precision medicine.^3^^,^^55^^,^^56^ Recognising these nuanced interactions will ensure more tailored and effective health interventions.

When designing health interventions that support changes in attitudes and behaviours related to rigid gender norms and stereotypes, a recent review concluded that design studies that involved groups with mixed gender identities were generally lower in quality than those working with single gender identity groups.^11^ Furthermore, this study stated the importance of dismantling and avoiding the reinforcement of rigid gender stereotypes during participatory sessions in which they are being addressed. During the participatory sessions in our study, the single gender identity groups were effective in avoiding the reinforcement of rigid norms and stereotypes due to the different perspectives that were shared within the men and women groups. This offered an exchange of diverse within-group experiences that contributed to an atmosphere of ‘*talking about*’ rather than ‘*talking from*’ norms and stereotypes. The general average Gender Self-Confidence score of the participants might also have contributed to this atmosphere. Although participants were allocated to the focus groups based on their Hoffman Gender Scale score, the within-group score distribution was relatively low, with few significant extremes. When people do not strongly associate their sense of self with masculinities or femininities, their perceptions and behaviours are less likely to be strongly regulated by cognitive ‘*gender schemas*’: the extent to which participants consider gender an important frame of reference and inclination to regulate their perception and behaviours through self-stereotyping.^57^^,^^58^ It might therefore be easier to ‘*talk about*’ gendered experiences because personal experiences are less processed and evaluated through the lens of normative gendered practices. We recommend further investigation into the moderating effects of the perceived salience of individual gender identity on the performance of gender norms and their impact on health outcomes for people with PD.

Besides the investigation of gender norms and stereotypes in personal illness experiences, it is equally important to investigate the gendered social systems that reproduce these experiences to ultimately address broader harmful social gender norms.^59^ For qualitative researchers studying gender stereotypes and norms, there is serious potential for a ‘catch-22’ situation when we are not aware of the ‘*talking about*’ versus ‘*talking from*’ social dynamics. In attempts to address gender biases in health, e.g., by making gender stereotypes explicit during focus group discussions, a mutual reinforcing effect can occur in which gender stereotypes are not only activated to ‘*talk about*’, but also become reinforced when participants ‘*take on*’ (stereotypical) roles that reproduce rather than deconstruct the gender biases researchers aim to address. When qualitative research results are then anecdotally reported without noticing this distinction in the social dynamics of the research context, researchers risk reproducing these gender biases as inevitabilities in patient experiences, rather than as social constructions within patient experiences. Consequently, researchers can, unconsciously, contribute to maintaining perceived gender norms through health research rather than reflecting critically on them. We consider substantial theoretical grounding in gender studies, strong listening and prompting skills of facilitators as key in supporting the investigation of gender as a social construct in health and illness experiences. Furthermore, including an instrument to measure how strongly participants associate themselves or internalise the gender dimensions under investigation, can support participatory workshop preparations.

## Contributors

IG contributed to the design and conceptualisation of the study, data collection and verification, data analysis, interpretation of data, drafting the manuscript and revising the manuscript for intellectual content. LM contributed to the design and conceptualisation of the study, data collection and verification, data analysis, interpretation of the data and revising the manuscript for intellectual content. PV contributed to the design and conceptualisation of the study, data collection and revising the manuscript for intellectual content. SOP contributed to the design and conceptualisation of the study, interpretation of the data and revising the manuscript for intellectual content. SKLD contributed to the design and conceptualisation of the study and revising the manuscript for intellectual content. BRB contributed to the revising the manuscript for intellectual content. All authors had access to the study data and accept responsibility for the decision to submit this manuscript for publication. No medical writer or editor was involved in the creation of this manuscript.

## Data sharing statement

The dataset used and analysed during the current study is not publicly available due to the sensitivity of the data (i.e., transcribed, and anonymised interviews with patients) and is available from the corresponding author upon reasonable request.

## Declaration of interests

The authors declare that they have no conflict of interest.
