# Supplementary material for: Gender-aware Parkinson’s care: a design-based study of patient perspectives on gender norms and gender-sensitive care
Source: eClinicalMedicine. 2023 Oct 17;65:102285. doi: 10.1016/j.eclinm.2023.102285 (PMC10590864; doi:10.1016/j.eclinm.2023.102285)
Supplement: Supplementary Material [file mmc1.pdf]

Table of content

Supplement 1. Overview and description of the included design phases and methods. --------------- 2

Supplement 2: Recommended elements for reporting of global health research that has used design -- 3

Supplement 3. The Hoffman Gender Scale --- 4

Supplement 4. The Nijmegen Gender Awareness in Medicine Scale --------------------------------------- 5

Supplement 5. Interview Guide Focus Group Discussions -- 7

Supplement 6. Post-Workshop Evaluation Questions for facilitators -------------------------------------- 8

Supplement 7. Operationalization of the self-reflective methods -- 9

Supplement 8. Pre-workshop survey | Hoffman Gender Scale | Open Question | Participants' definitions of masculinity and femininity. --- 10

Supplement 9. Self-reflective methods | Reverse Thinking Results -------------------------------------- 12

Supplement 1. Overview and description of the included design phases and methods.

**Included phases of the equity-centered design process, applied methods and objectives**			
**Phase**	**Description**	**Applied methods**	**Methodological objective**
**Empathy**	The EMPATHY phase of the process is focused on understanding the experiences, emotions and motivations of men and women with Parkinson's disease (PD). In this phase we aim to uncover gender stereotypes and gender norms that men and women with PD experience in their daily lives and how PD impacts these stereotypes or norms and vice versa.	Hoffman Gender Scale.<br><br>Nijmegen Gender Awareness Scale<br><br>Focus Group Discussions	To assess how strongly committed participants were to their gender identity to allocate participants to FGD subgroup (higher vs lower committed).<br><br>To assess the degree to with participants were sensitive towards the role of gender in medical care as a baseline awareness measure towards the topic.<br><br>To explore and identify gender norms or stereotypes present in illness experiences of men and women with PD.
**Define**	The DEFINE phase of the process if focused on developing a point of view about the needs of men and women with PD with regards to gender-aware PD care. During this stage of the process, data from the first phase will inform the design challenge: "How might clinical care for men and women with Parkinson's Disease become more gender aware?"	Reverse/Inverted Thinking<br><br>Word-Concept Association<br><br>Download the Learning	Warm-up exercise towards insights by flipping the logic of ideation and stoke creativity.<br><br>To cluster perceptions and prioritize design features and concepts.<br><br>To formulate individual key learnings, impressions, and experiences.
**Ideate**	The IDEATE phase of the process is focused on the generation of as many solutions to the design question(s) as possible. Once many solutions have been generated, participants will select and prioritize key ideas that could move forward to prototyping.	Collective insight statements	To share individual learnings and make them part of the collective groups' knowledge base to brainstorm recommendations for the design question: '*how might we design gender aware PD care?*'
**Notice & Reflect**	The NOTICE & REFLECT phase of the process is ongoing and transparent throughout the design process. It allows the research team to take time to focus and reflect on actions, emotions, insights and impacts as researcher and as humans. This phase activates cultural and social emotional awareness within and among the research team before entering the empathy phase, to allow an authentic human-centered design practice	Reflective discussions<br><br>Post-workshop evaluation	To cultivate awareness among the facilitation team regarding the sensitivity of the topic, workshop design and research context.<br><br>To deliberately center principles of inclusion and equity centered design and think about their role and relevance during the participatory design session and the data analyses.

Supplement 2: Recommended elements for reporting of global health research that has used design

Item	Paper section	Topic/descriptor
1	Title and abstract	Title should indicate that the study included a design approach. Abstract summarises the salient components, including background, statement of problem, approach/methods, findings, results and conclusion.
2	Introduction/background	Overview of the background to the topic, what has been done in the area already and rationale for using design. Description of the initial research question or health problem that the design-based work aimed to address.
3	Methods/approach	Reporting of the approach, tools or processes that were used for the research; who was involved (both researchers and participants); when and where research was conducted; how data were reviewed, analysed and synthesised; and iteration.
4	Results/findings from design research and activities	Summary of findings from design activities. Explanation of what was finally designed and what the associated decision-making points were, and (if available) impact. Report on any secondary or ancillary results.
5	Discussion	Reflection on the incorporation of design to this research topic, including but not limited to strengths, limitations and contribution.
6	Conclusion	Implications of this work for the larger fields of health and design, and any next steps.
7	Other	Ethical considerations, relevant acknowledgements of conflict of interest, funding, and contributorship of authors, designers, stakeholders and participants.

Bazzano AN, Yan SD, Martin J, et alImproving the reporting of health research involving design: a proposed guidelineBMJ Global Health 2020;5:e002248.

Supplement 3. The Hoffman Gender Scale

**Hoffman Gender Scale: Measurement for Gender Self Confidence**	
Subscale	GSD = Gender Self-Definition subscale (how salient gender is to individual identity)<br><br>GSA = Gender Self-Acceptance subscale (how comfortable a person is as a member of his/her gender)<br><br>Rating 1 = sterk mee oneens, 6 = sterk mee eens)
GSD1	Als aan mij gevraagd wordt om mezelf te beschrijven, is man-zijn/vrouw-zijn een van de eerste dingen waar ik aan denk.
GSA1	Ik ben zelfverzekerd in mijn mannelijkheid/vrouwelijkheid.
GSA2	Ik voldoe aan mijn persoonlijke standaard voor mannelijkheid/vrouwelijkheid.
GSD2	Mijn perceptie van mijzelf is positief geassocieerd met mijn biologisch geslacht.
GSA3	Ik voel me zeker in mijn mannelijkheid/vrouwelijkheid.
GSD3	Ik definieer mijzelf grotendeels in termen van mijn mannelijkheid/vrouwelijkheid.
GSD4	Mijn identiteit is sterk verbonden met mijn mannelijkheid/vrouwelijkheid.
GSA4	Ik heb veel waardering voor mijzelf als man/vrouw.
GSD5	Man-zijn/vrouw-zijn is een cruciaal onderdeel van hoe ik mezelf zie.
GSA5	Ik ben blij met mijzelf als man/vrouw
GSA6	Ik voel me erg op mijn gemak als man/vrouw
GSD6	Mannelijkheid/vrouwelijkheid is een belangrijk aspect van mijn zelfbeeld.
GSA7	Mijn gevoel van mezelf als man/vrouw is positief
GSD7	Man-zijn/Vrouw-zijn draagt voor een groot deel bij aan mijn gevoel van zelfvertrouwen.
Open Question	Wat verstaat u onder mannelijkheid/vrouwelijk?

For English version, see: Hoffman, R.M. & Borders, L. & Hattie, John. (2000). Reconceptualizing femininity and masculinity: From gender roles to gender self-confidence. Journal of Social Behavior and Personality. 15. 475-503.

Supplement 4. The Nijmegen Gender Awareness in Medicine Scale

**Nijmegen Gender Awareness Scale**	
Subscale	Gender Sensitivity: In hoeverre bent u het eens met de onderstaande stellingen (1 = helemaal mee oneens, 5 = helemaal mee eens)
GS1_R	Artsen moeten alleen rekening houden met biologische verschillen tussen mannen en vrouwen.
GS2_R	Bij niet-geslachtsgebonden aandoeningen is het geboortegeslacht van de patiënt niet belangrijk.
GS3_R	Een arts moet zich zoveel mogelijk beperken tot medische aspecten van klachten van mannen en vrouwen.
GS4_R	Verschillen tussen mannelijke en vrouwelijke artsen zijn te klein om relevant te kunnen zijn.
GS5_R	Juist omdat mannen en vrouwen verschillend zijn moeten artsen iedereen op dezelfde wijze behandelen.
GS6_R	Artsen die rekening houden met geslachtsverschillen houden zich niet met de belangrijke zaken bezig.
GS7_R	In de communicatie met patiënten maakt het voor een arts niet uit of het mannen of vrouwen zijn.
GS8_R	In de communicatie met patiënten maak het voor de arts niet uit of de arts zelf een man of een vrouw is.
GS9_R	Verschillen tussen mannelijke en vrouwelijke patiënten zijn zo klein dat artsen er nauwelijks rekening mee kunnen houden.
GS10_R	Rekening houden met verschillen tussen mannen en vrouwen veroorzaakt ongelijkheid in de gezondheidszorg.
GS11_R	Het is niet nodig om rekening te houden met verschillen in de wijze waarop mannen en vrouwen hun gezondheidsklachten presenteren.
GS12	Kennis over geslachtsverschillen in ziekte en gezondheid bij artsen verhoogd de kwaliteit in de gezondheidszorg.
Subscale	Gender role ideology towards patients: Bent u van mening dat (1 = helemaal mee oneens, 5 = helemaal mee eens)
GRIP1	Mannelijke patiënten de werkwijze van artsen beter begrijpen dan vrouwelijke patiënten.
GRIP2	Vrouwelijke patiënten onnodig veel van artsen vragen vergelijken met mannelijke patiënten.
GRIP3	Vrouwen vaker dan mannen met de arts problemen willen bespreken die niet thuishoren in de spreekkamer.
GRIP4	Vrouwen te veel emotionele steun van artsen verwachten.
GRIP5	Mannelijke patiënten minder veeleisend zijn dan vrouwelijke patiënten.
GRIP6	Vrouwen meer gebruik maken van de gezondheidzorg dan werkelijk nodig is.
GRP7	Mannelijke niet naar een arts gaan voor onschuldige gezondheidsproblemen.
GRIP8	Medisch onverklaarbare aandoeningen bij vrouwen ontstaan doordat zij teveel zeuren over gezondheid.
GRIP9	Vrouwelijke patiënten over hun gezondheid klagen omdat ze meer aandacht nodig hebben dan mannelijke patiënten.
GRIP10	Het gemakkelijker is om oorzaken te vinden van klachten bij mannen omdat mannen gewoon zeggen waar het op staat.
GRIP11	Mannen vaker dan vrouwen een beroep doen op de gezondheidszorg met problemen die zij hadden moeten voorkomen.
Subscale	Gender role ideology towards doctors: Bent u van mening dat (1= helemaal mee oneens, 5 = helemaal mee eens)
GRID1	Mannelijke artsen te veel nadruk leggen op technische aspecten van de geneeskunde vergeleken met vrouwelijke artsen.
GRID2	Vrouwelijke artsen vergelijken met mannelijke artsen hun consulten te veel laten uitlopen.
GRID3	Mannelijke artsen efficiënter zijn dan vrouwelijke artsen
GRD4	Vrouwelijke artsen empathische zijn dan mannelijke artsen

GRID5	Vrouwelijke artsen onnodig veel rekening houden met de belevingswereld van de patiënt.
GRID6	Mannelijke artsen zijn gehaaster in hun werk vergeleken met vrouwelijke artsen.
GRID7	Vrouwelijke artsen emotioneel meer betrokken zijn bij hun patiënten dan mannelijke artsen.

For English version, see: Verdonk, Petra & Benschop, Yvonne & Haes, Y. & Lagro-Janssen, Antoinette. (2008). Medical students’ gender awareness: construction of the Nijmegen Gender Awareness In Medicine Scale (N-GAMS). Sex Roles. 58. 222-234.

Supplement 5. Interview Guide Focus Group Discussions

Domains	Topic
Gender Identity	- Can you talk a little about the perspectives and ideas you grew up with regarding being male/female? How do you view it now as an adult?<br>- What do terms like "masculinity" and "femininity" mean to you?<br>- How would you describe your own gender identity? Is your being male or female an important part of your identity?<br>- Do you consider yourself a 'traditional' man/woman? What does this mean to you?<br>- Are there specific activities that are an important expression of your being male or female? What role does Parkinson's disease play in this for you?
Gender Norms and Stereotypes	- In your opinion, what is the general perception of men/women with Parkinson's?<br>- In your opinion, are there any stereotypical images or ideas about men/women with Parkinson's? Do you recognize yourself in these images, why/why not?<br>- Are there certain social expectations in your environment that you as a man/woman should meet according to others? Who are those 'others'?<br>- How do you deal with these expectations? Does Parkinson's disease play a role in this for you? Is it hindering or helpful?
Gender Sensitive Care	- What do you think of when you think of "gender sensitive care for people with Parkinson's"?<br>- What do you think of when you think of more sensitive care for men/women with Parkinson's?

Supplement 6. Post-Workshop Evaluation Questions for facilitators

<table>
<tr><td></td><td>Post-workshop reflective questions</td></tr>
<tr><td>1</td><td>Based on your own observations, (briefly and intuitively) formulate an answer to the primary research question?</td></tr>
<tr><td>2</td><td>Based on your own observations, (briefly and intuitively) formulate an answer to the primary design question?</td></tr>
<tr><td>3</td><td>Was the focus group method an effective method to explore gender norms in illness experiences of men and women with PD?</td></tr>
<tr><td>4</td><td>Optional: Which methods might be (even) more effective to explore gender norms in illness experiences? How might we explore them more effectively (=more informatively)?</td></tr>
<tr><td>5</td><td>Was the structured brainstorm an effective method to explore concepts for gender-sensitive care for men and women with PD?</td></tr>
<tr><td>6</td><td>Optional: Which methods might be (even) more effective to explore concepts for gender-sensitive PD care? How might we explore them more effectively (=more informatively)?</td></tr>
<tr><td colspan="2">Equity Centered Design - Notion & Reflect Phase

Reflect on the following principles and think about their role and relevance during the workshop and upcoming data analyses.

Principles of Inclusive and Equity Centered Design:

Recognize Exclusion
Checking personal biases, including those around disabilities and related limitations, to avoid conscious or unconscious exclusionary decisions.

Learn from Diversity
Letting research insights be driven by the unique perspectives of diverse individuals and the way they adapt to experiences not originally designed for them.

Solve for One, Extend to Many
Focusing on what's universally important to all humans and understanding the power of solving along the continuum of permanent disabilities to temporary disabilities (e.g., broken arm) to situational impairments (e.g., loud crowd affecting your hearing).

Design at Margins
Building for marginalized communities who are most hurt by oppression, and bringing them into the design process.

Start with Self
Recognizing personal mental models, including how biases and assumptions impact solution design on both a conscious and unconscious level.

Cede Power
Providing power to underrepresented individuals that are brought into the design process, and making it a safe space for speaking truth to injustices.

Make the Invisible Visible
Recognizing, explicitly calling out, and actively challenging hegemonic practices that have historically advantaged dominant groups over marginalized groups.

Speak to the Future
Finding new language to complement the design of a new, equitable future, such as defining an innovation as an increase in equity and reduction of racism.</td></tr>
<tr><td>7</td><td>What social demographics should we be aware of in our study population? Who might we be excluding?</td></tr>
<tr><td>8</td><td>How could identities within our team (have) influence(d) or impact(ed) data collection and data analysis decisions? What do we need to be aware of moving forward towards the data analysis?</td></tr>
<tr><td>9</td><td>During data analysis: How can we ensure we are focussing on an actual need or question that this community has, rather than one we may be incorrectly perceiving they have (implicit/explicit biases)?</td></tr>
<tr><td>10</td><td>During data analysis: How can we ensure we are focussing on an actual need or question that this community has, rather than one we may be incorrectly perceiving they have (implicit/explicit biases)?</td></tr>
</table>

Supplement 7. Operationalization of the self-reflective methods

Method	Operationalization
Reverse Thinking	How would ‘gender sensitive care’ for people with Parkinson’s look like if we would design it completely wrong?
Word-Concept Association	Write down words that you associate with “care for women/men with Parkinson’s”.
Downloaded Learning	Finish these sentences:<br><br>For me, care for women/men with Parkinson's means paying attention to:…….(so that/because)………<br><br>Attention to this care is (mainly) important/relevant when:….

Supplement 8. Pre-workshop survey | Hoffman Gender Scale | Open Question | Participants' definitions of masculinity and femininity.

What do you understand by 'masculinity'?*	
#	Response
1	Being part of society, regardless of whether you are male or female
2	The influence of my hormones determines my behavior and will not be influenced by concepts such as "Masculinity", which is relative. How can I know what I would feel with a higher testosterone level or estrogen?
3	Role distributions between male and females
4	That you are assertive and know what you stand for
5	Tough question. Not being able to get pregnant.
6	Less emotional, more rational, loves women, physically stronger, together with woman a good couple
7	That I was born a man
8	The fact, that I have an outward appearance of a man....
9	Radiate that you are full of self-confidence
10	Still to be determined
11	Masculinity for me is being self-reliant, tough, and strong. Strong in muscular strength but also in decisiveness and decisiveness. Masculinity for me is also being sexually active, not showing too many emotions and being successful in your work.
12	Showing male behavior
13	Primarily related to sex. Other traits, in my view, are more related to the personality
14	Biological characteristics of the male
15	So called here, all this feels like a very old-fashioned proposition: the man strong, tough, not faint, leading. I have seen that for a very long time: man -woman=equal. Being a man is a given, but being human is the basis.
16	Healthy attention to female beauty!
17	Cannot be said in three words
18	Biologically determined and society-influenced set of behavioral characteristics
19	Stand by what you say or do. A man a man, a word a word! Straightforward and in most cases do not step aside. Warning once should be enough. Respect all women and treat them the same. Don't participate in gossip etc. Take care of those you love.
20	Strongly inquisitive, protective, gallant, camaraderie.

*Responses of male participants.

What do you understand by 'femininity'?*	
#	Response
1	The physique, the actions, the ideas, the solutions, the thinking, the appearance, the organization, the strength, the structuring
2	The female sex, motherhood, feminine appearance, caring,
3	Difficult question - but is about empathy, caring, being confident in what I do. Being proud of my children, doing my part for a living, male characteristics are also part of me, winning.

4	Femininity for me consists of motherliness, gentleness, empathy, seeing connections, seeing interests of different groups or family members. Being able to connect and behave that way. Not being submissive but being strong. Setting boundaries. Being attractive, seductive if desired, authentic.
5	Empathy, sensitivity, emotional
6	Sex Motherhood Softness
7	Just being yourself and radiating that
8	A combination of purely biological, external, and genetic set of differences from men and, in addition, cultural and social expectations regarding being a woman. I struggled a lot with the above questions. I see myself as human, I am a woman and was very happy to become a mother but don't feel particularly feminine and am not much about it. Although I find it very annoying when I notice that I or other women are disadvantaged by being a woman.
9	That you appreciate who you are and what you put out.
10	Characteristics and behaviors and roles that typically belong to women such as choosing clothes and professions and behaving in a dependent (vulnerable) manner, among others.
11	I feel completely female because of how I look. How people respond to me. I just can't imagine how to feel like a man. I'm just a woman. And feel very comfortable under that.
12	Caring, independence, cleverness combined with inner beauty, versatility, justice and being sensitive.
13	By that I mean that we as women are allowed to have children. That we are given curves and more extras to distinguish us from men.
14	Caring and compassionate....

*Responses of female participants.

Supplement 9. Self-reflective methods | Reverse Thinking Results

Gender identity category	How would 'gender sensitive care' for people with Parkinson's look like if we would design it completely wrong?
Men	- Assuming that all men are equal. No longer paying attention to issues that are important in men.<br>- Not asking questions. Making assumptions.<br>- Anything that goes too fast and creates stress; giving the impression of being in a hurry.<br>- Not taking into account differences between men and women; differences in interest and also differences in age due to the presence of children.<br>- Probably achieve less effect and also coping will be less good. The influence of social influence will be decisive.<br>- Assume dependency. Think in terms of problems. Especially dwell on the progression of Parkinson's disease. Not naming your own contribution to the conversation/ coping with Parkinson's disease. Avoiding certain topics, such as work/hobby/volunteer work/sports/emotions/sexuality. Not naming any resources.<br>- No approach. Falling back on past approaches? No examination of medication.<br>- No attention to social problems, no attention to addiction.<br>- No attention to feelings and emotions. No attention to sexual limitations. No attention to performance/sports. No attention to attention, actual attention to anxiety(s).<br>- Risk of wrong treatment. For example, in cardiology, for a very long time there was no distinction between men and women. Result is wrong treatment of cardiac issues in women. Wrong interpretation of symptoms and thus treatment of Parkinson's disease.<br>- No attention to the emotional aspect and no attention to the importance of exercise (walking, cycling, fitness).<br>- Possibly then a (healthcare) model is created in which only sex (biological) is distinguished. I consider this risk quite high because no other classification with corresponding relevant research data is known. By the way, this need not be COMPLETELY wrong.<br>- Care would then not take into account my 'being a man'. 'Parkinson's' is different for everyone, so all-encompassing care will/cannot suffice.
Women	- I expect that wrong medication will be prescribed and there will be many more complaints among women.<br>- More self-neglect, informal care.<br>- Maybe the way it is now? If it does matter it goes wrong (now). If it doesn't matter, it doesn't matter and nothing changes in health care providers. Caregivers probably work more randomly if they are not aware of possible gender influences.<br>- Assuming that care for men with P also applies to women. Not taking into account differences such as hormone changes. No other advice to women in terms of lifestyle, medication, nutrition, in relation to being a woman. Offering male-oriented care to women and assuming that women react the same way to medication etc. Not listening to the specific complaints of the woman. "I don't have an answer" and not being referred for woman specific questions.<br>- Not taking into account the difference between men and women This should not be static, it is not black and white.<br>- Using stereotypes to e.g. approach women only empathetically, to educate men only cognitively. Caregivers know what is good for the patient - don't check in, don't consider grief, communicate from negativity "just enjoy the opportunities you still have".<br>- Women not being "read" properly and therefore receiving less/no proper counseling. Perhaps an anti-neurology movement arises against levodopa or other medications making the "followers" worse off. The relationship "thinking for yourself" is punished -- not good. Women are not taught, actively thinking and feeling -- not good for them. Transition -- important. Changes not being included.<br>- Not listening to the women or man affected, applying male research findings 1-1 to women, no understanding of hormones + parkinson's, no consideration of transition/child desire, no attention to female sexuality, treating emotions as female.<br>- Working in health care from stereotypical thoughts. Not questioning complaints and symptoms. Sticking to a non-holistic attitude towards patients (leads to less stereotyping??). I myself was treated by 4 specialties but no one had a total overview except myself. As a result, I tried very hard to put complaints in a broader perspective. Medicine is too much divided into specialties, possibly a disadvantage for the Parkinson's patient -- a Parkinson's specialty.<br>- I assume that this concern can never develop completely wrong. If it did that would be solvable through communication. Caregivers can also learn from others in consultation and conclude from that to work with new methods.<br>- Assessing/questioning men and women the same way. Making assumptions. Not asking experts by experience. Not involving immediate family. Fixed / procedure-driven investigations (not being open to conflicting (gender) perceptions). Not listening. Subordinating.<br>- Prescribing medication according to a fixed protocol starting too high instead of building up slowly. Generic explanation of illness with too few solutions about other treatment options. What health care providers do: stigmatize, trivialize, medicate too quickly. Do not: ask questions, be empathetic, present alternatives, emphasize the importance of exercise and daring to ask for help. Point.

	• Then they start filling in for you what would be good. I am no longer taken seriously and assumptions are made that are not helpful. For me then the right medication does not apply. They start being authoritarian and then I become recalcitrant and then I languish behind the geraniums.<br>• Then women would not get the chance to develop their image regarding their illness.<br>• Little/no attention to emotion: what does the diagnosis do to you, what does the disease do to you. Insensitive handling of input on patient complaints. Not seeing women as equal. Quickly naming complaints as nagging or exaggerated. Cooler approach to the patient.
